# Streptococcal Adhesin P (SadP) contributes to *Streptococcus suis* adhesion to the human intestinal epithelium

**DOI:** 10.1371/journal.pone.0175639

**Published:** 2017-04-13

**Authors:** Maria Laura Ferrando, Niels Willemse, Edoardo Zaccaria, Yvonne Pannekoek, Arie van der Ende, Constance Schultsz

**Affiliations:** 1 Department of Medical Microbiology, Center for Infection and Immunity, Academic Medical Center, University of Amsterdam, Amsterdam, The Netherlands; 2 Department of Global Health-Amsterdam Institute for Global Health and Development, Academic Medical Center, University of Amsterdam, Amsterdam, The Netherlands; 3 Host-Microbe Interactomics, Animal Sciences, Wageningen University, Wageningen, The Netherlands; Oregon Health & Science University, UNITED STATES

## Abstract

**Background:**

*Streptococcus suis* is a zoonotic pathogen, causing meningitis and septicemia. We previously demonstrated that the gastrointestinal tract (GIT) is an entry site for zoonotic *S*. *suis* infection. Here we studied the contribution of Streptococcal adhesin Protein (SadP) to host-pathogen interaction at GIT level.

**Methods:**

SadP expression in presence of Intestinal Epithelial Cells (IEC) was compared with expression of other virulence factors by measuring transcript levels using quantitative Real Time PCR (qRT-PCR). SadP variants were identified by phylogenetic analysis of complete DNA sequences. The interaction of SadP knockout and complementation mutants with IEC was tested *in vitro*.

**Results:**

Expression of *sadP* was significantly increased in presence of IEC. Sequence analysis of 116 invasive strains revealed five SadP sequence variants, correlating with genotype. SadP1, present in zoonotic isolates of clonal complex 1, contributed to binding to both human and porcine IEC and translocation across human IEC. Antibodies against the globotriaosylceramide Gb3/CD77 receptor significantly inhibited adhesion to human IEC.

**Conclusion:**

SadP is involved in the host-pathogen interaction in the GIT. Differences between SadP variants may determine different affinities to the Gb3/CD77 host-receptor, contributing to variation in adhesion capacity to host IEC and thus to *S*. *suis* zoonotic potential.

## Introduction

*Streptococcus suis* (SS) is an emerging zoonotic pathogen which can cause severe disease including meningitis and septic shock in human and pigs [1]. The capsular polysaccharide (CPS) is a virulence factor of *S*. *suis* which determines the serotype; the virulence and prevalence differ within and among serotypes [2]. In addition to serotype, the genotype contributes to virulence of *S*. *suis* [3, 4]. Invasive *S*. *suis* strains are limited to certain sequence types (ST), as determined by multi locus sequence typing (MLST). *S*. *suis* serotype 2 (SS2) isolates belonging to MLST clonal complex 1 (SS2/CC1) are considered highly virulent and zoonotic [2]. In the Netherlands, SS2 isolates belonging to MLST clonal complex 20 (SS2/CC20), also contribute to human disease [4]. In contrast, *S*. *suis* serotype 9 (SS9), part of MLST clonal complex 16 (SS9/CC16) and the main cause of porcine *S*. *suis* infections in Northern Europe, is rarely associated with human infection [4, 5]. Therefore, CC1, CC16 and CC20 are most common virulent genotypes circulating in the Netherlands.

Epidemiological and experimental studies indicated that SS2/CC1 infection is a foodborne disease in Southeast Asia in adult patients caused by the consumption of contaminated undercooked pork [6–9]. So far, it still unknown which bacterial factors contribute to potential risk of *S*. *suis* zoonotic transmission through the *S*. *suis* translocation of the human gastrointestinal tract.

We previously demonstrated that SS2/CC1 adhered to and translocated across intestinal epithelial cells (IEC) of both human and porcine origin *in vitro*, and translocated the gastrointestinal tract (GIT) in a piglet model after *S*. *suis* oral infection [6]. The capacity of *S*. *suis* isolates to adhere to human and porcine IEC correlated with serotype as SS9 isolates showed significantly less binding to human IEC than SS2 isolates, suggesting that CPS composition and structure mediate host species restriction. In addition, whilst the CPS was shown to prevent adhesion to host-cells in *in vitro* models [6, 10], unencapsulated *cps* mutant strains of serotype 2 and 9 appeared to retain the host restriction of their encapsulated parental strains when interacting with human and porcine IEC [6]. Thus, in addition to the capsule, secreted or cell-associated virulence factors such as adhesins are likely to contribute to host-specific interactions of *S*. *suis* with IEC.

Streptococcal adhesin P (SadP) is a cell-wall adhesin which can recognize galactosyl-α1-4galactose (Galα1-4Gal or galabiose) [11] present as the terminal epitope of globoseries glycolipids (Gbs) receptors on erythrocytes [12]. Analysis of SadP binding specificity to galabiose present in the Gbs receptors revealed the highest specificity to the globotriaosylceramide Gb3/CD77 receptor [11]. Gb3/CD77 receptors are abundant in various host tissues including human and pig small intestine in the precursor (Gb3/CD77) or mature form (globotetraosylceramide Gb4) [13, 14]. Furthermore, Gb3/CD77 is a receptor for adhesin-mediated binding to host tissues of multiple human pathogens and serves as receptor for bacterial toxins (Shiga toxins, Stx) [15].

First, we determined the relevance of SadP in adhesion to intestinal epithelial cells in comparison to other well-characterized virulence-associated factors. Therefore, we assessed transcription levels in *S*. *suis* in the presence or absence of intestinal epithelial cells of well-characterized *S*. *suis* secreted or cell-associated factors selected on the basis of their functional role in bacterial interaction with the host epithelium [16]. The differential transcription of *sadP* was highest among eight virulence-associated factors. We hypothesized that SadP plays a dominant role in the interaction of *S*. *suis* with porcine and human intestinal epithelium, and we performed a comparative study of SadP mediated adherence of *S*. *suis* serotype 2 (belonging to CC1 and CC20) and 9 (belonging to CC16).

## Materials and methods

### Bacterial strains and intestinal cell lines

Bacterial isolates used are listed in **Table A in S1 File**. All strains were grown in Todd-Hewitt Broth with 5% Yeast (THY) or on Columbia blood agar plates (Difco). *E*. *coli* BHB2600 [17] was cultured in Luria-Bertani (LB) broth or on LB agar (Difco). When necessary, antibiotics were added to culture media at the following concentrations: for *E*. *coli* 50 mg/L spectinomycin (s*pc*); for *S*. *suis* 200 mg/L kanamycin (*kan*) and 100 mg/L s*pc*. Caco-2 cells (HTB-37™, ATCC) and IPEC-J2 cells (ACC 701, DSMZ) were grown as previously described [18, 19].

### RNA extraction from co-culture of *S*. *suis* and IECs

Caco-2 and IPEC-J2 cells were cultivated in triplicate in T25 flasks until differentiation [6]. Co-culture of IEC-bacteria was conducted without antibiotics and Fetal Calf Serum (FCS). The bacterial suspension (~ 50 bacteria/cell Multiplicity Of Infection [MOI]) was added to flasks with IEC and without IEC (control) and incubated for 4 hours at 37°C in 5% CO_2_. The co-culture of bacteria and IECs was stopped by adding ice-cold 95% ethanol/5% phenol, pelleted, and stored at -80°C [20]. Total RNA was extracted using the hot-phenol method [20] followed by RNA purification with miRNeasy Minikit (Qiagen). Purified RNA was treated with TURBO DNA-*free™* (Life Technologies). Quantity and quality of the RNA were measured with NanoDrop 2000 (Thermo Scientific) and Bioanalyzer (Agilent technologies).

### Quantitative real time PCR (qRT-PCR)

Reverse transcription of the RNA was performed with ThermoScript rt-PCR (Invitrogen). Relative gene expression was determined by qRT-PCR using SYBR Green (Roche) using two housekeeping genes (*proS* and *gdh*) as reference genes [21]. All samples were run in biological triplicates. Primers are listed in **Table B in S1 File** and data were analyzed with LinRegPCR [22].

### MLST and whole genome sequencing

The genotype of the strains was previously determined by MLST [4]. DNA isolation, library preparation, sequencing, and assembly were previously described in detail [23]. Briefly, *S*. *suis* genomic DNA was extracted and fragmented by sonication [23]. Sequencing libraries were created using an in-house protocol and paired-end sequencing was performed on the Illumina MiSeq sequencing platform [23]. The reads were trimmed with CutAdapt and Sickle [24] and assembled with SPAdes 3.0 [25].

### Phylogenetics

Whole genome sequences (WGS) of 116 *S*. *suis* isolates were mined for the presence of SadP using NCBI's BLAST. Protein SSU0253 from strain P1/7 was used as query SadP sequence against a protein database, generated using Prodigal [26] predicted protein sequences from the draft genomes. The e-value was set at 1e-5 and proteins with >50% identity over >50% of the alignment were identified as SadP proteins. The corresponding nucleotide sequences of the 111 strains that were shown to contain SadP were aligned at the protein level using MUSCLE [27], and a phylogenetic tree was constructed using PhyML [28] with 100 bootstraps. An extended phylogenic analysis included 375 additional publicly available WGS of *S*. *suis* strains from the UK and Vietnam [29]. Prodigal was used to predict coding sequences in this dataset.

### Construction of *S*. *suis sadP* deletion mutants

Primers used for mutagenesis are listed in **Table B in S1 File**. *sadP* deletion was achieved by gene replacement with the Janus cassette [30] carrying a *kan* gene. PCR fragments containing the flanked region of *sadP* (~ 0.5 kb) and Janus cassette were digested with ApaI/BamHI or EcoRI/BamHI and then ligated. Δ*sadP*::*Janus* fragment was PCR amplified from the ligation using Phusion DNA Polymerase (Thermo Fisher) with external primers, and successively transformed into *S*. *suis* in presence of the competence-inducing peptide ComS13-21 as previously described [31]. Transformants were selected on Columbia agar plates with kanamycin. The insertional mutagenesis of Δ*sadP*::*Janus* was confirmed by PCR and sequencing.

### Complementation of deletion mutants

The pMX1 vector [32] was used for the generation of complementation mutants in strain 10Δ*sadP1* (**Table A in S1 File**). The plasmid was maintained into *E*. *coli* BHB2600 kindly provided by Dr. S.A.J. Zaat [17] and extracted using GeneJET Plasmid Midiprep (Thermo Fisher). The complete *sadP1*, *sadP2A*, and *sadP2B* genes were amplified from genomic DNA of respective *S*. *suis* strains (**Table B in S1 File**) and cloned into pMX1 via EcoRI/BamHI sites. After ligation, the plasmid was directly introduced into 10Δ*sadP1* in presence of ComS13-21 to construct 10Δ*sadP1*C*sadP1*, 10Δ*sadP1*C*sadP2A* and 10Δ*sadP1*C*sadP2B* complemented mutants.

### Adherence and translocation assays using IECs

Adhesion and translocation assays were performed as previously described [6]. For inhibition of bacterial adhesion assays, Caco-2 cells were pre-incubated with increasing concentrations of monoclonal anti-Gb3/CD77 (Biocompare) in medium containing FCS, for 1 hour at 37°C, after which bacteria were added to the cells. Buffer (PBS, 0.1% sodium azide, 0.2% BSA) without antibody was used as a control.

### Statistical analysis

Prism 6.0 (GraphPad software, USA) was used to analyse the normalized qPCR fold-ratio to the control (no cells) by One-way ANOVA. Unpaired Student's *t*-test was performed to compare the percentage of adhesion to and translocation across IECs between 10Δ*sadP1* mutant and its parental strain.

## Results

### *SadP* gene expression increases in the presence of human and porcine IEC

**Fig 1:**
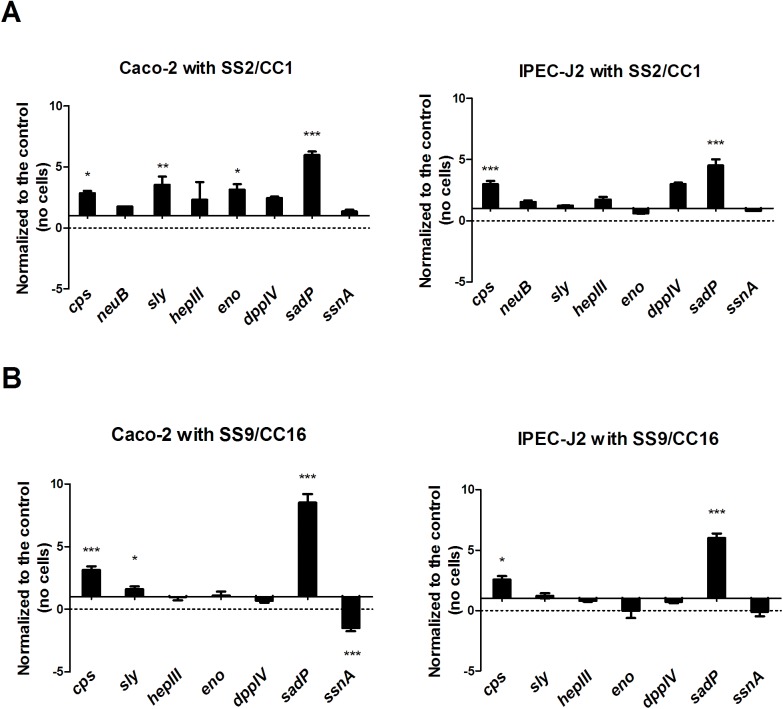
**Normalized differential gene expression of eight virulence factor genes during the interaction of SS2/CC1 strain 10 and SS9/CC16 strain 8067 with A) human (Caco-2) and B) porcine (IPEC-J2) IECs.** RNA was extracted from the adherent *S*S2/CC1 strain 10 and SS9/CC16 strain 8067 after 4 hour of co-incubation with IECs to quantify the expression level of known virulence genes by qRT-PCR. As a control experiment, gene expression was estimated in *S*. *suis strains* incubated under identical conditions without epithelial cells. The expression of each gene was normalized to that of the internal reference *proS* and *gdh* genes [19]. Relative expression levels were calculated as the expression of a gene divided by that in non-adherent *S*. *suis* at the end of the experiment (4h), which was arbitrarily defined as 1. Data presented are averages of three independent experiments, and error bars represent standard deviations. The significance of differences in the expression of the genes in non-adherent and adherent *S*. *suis* was determined by one-way ANOVA indicated as follows: (***, *p* < 0.001; **, *p* < 0.01; *, *p* < 0.05).

We first used qPCR expression profiling during host-pathogen protein interactions to identify whether *sadP* expression was upregulated in presence of human and porcine IEC as an indication of its importance in adhesion. Therefore, we compared *sadP* transcript levels in comparison with other well-characterized *S*. *suis* secreted or cell-associated factors selected on the basis of their functional role in bacterial interaction with the host epithelium [16] as described in **Table 1** [11, 33–40]. Transcript levels were determined in isolates representing zoonotic (*S*S2/CC1 strain 10) and non-zoonotic (SS9/CC16 strain 8067) *S*. *suis* types (**Table A in S1 File**), following 4 hours of co-culture in cell culture media without IEC (control) and with human (Caco-2) and porcine (IPEC-J2) IECs. Transcript levels of *sadP* in both *S*. *suis* strains ranged from 4.5 to 8.5-fold (*p*<0.001) higher than bacteria grown without IEC (**Fig 1**). Interestingly, in *S*. *suis* grown in the presence of IECs transcript levels of *cps* were also higher (2.5 to 3.0-fold *p*<0.05 and *p*<0.001 respectively) compared to *S*. *suis* grown without IECs. *Sly* toxin transcript levels were 1.6 to 3.5-fold higher (*p*<0.05) in both strains when in contact with human IEC only. In contrast, transcript levels of *ssnA* in SS9/CC16 in presence of IEC were 1.4 fold lower compared to control (*p*<0.001). Transcript levels of genes coding for the other virulence factors were not significantly altered compared to the control without cells except for transcript levels of the protease *ddp IV* involved in cell invasion process [16, 21], which was significantly upregulated in SS2/CC1 in presence of both IECs (~ 3.0-fold higher with Caco-2 *p*<0.05 and IPEC-J2 *p*<0.001). The high levels of *sadP* transcripts compared to other virulence factors, suggested an important role of SadP during *S*. *suis*-IEC interaction among zoonotic and non-zoonotic isolates.

**Table 1:** Selected virulence genes [16] for qRT-PCR gene expression analysis.

Annotation SS2/CC1 strain P1/7—SSU locus	Protein	Function	Interaction with host-epithelium	Virulence	Biblio
**Streptococcal adhesin P-SSU0253**	SadP	Binds to galabiose of Gbs^1^	Adhesion epithelium	No mutant	[11]
**Enolase-SSU1320**	Eno	Fibronectin-plasminogen binding	Adhesion ECM^2^	No mutant	[33]
**Di-peptidyl peptidase IV-SSU0187**	DppIV	Fibronectin binding	Adhesion ECM^2^	Attenuated-mouse	[34]
**HP0197-SSU1048**	Hp0197/ HepIII	GAG^3^ -heparin binding	Adhesion epithelium	Not tested	[35]
**Galactosyl/rhamnosyl transferase-SSU0520**	CpsE/F	CPS biosynthesis	Adhesion/Invasion	Attenuated-pig	[36]
**N-acetylneuramic acid synthase-SSU0535**	NeuB	Sialic acid synthesis	Adhesion/Invasion	Attenuated-pig	[37]
**Suilysin—SSU1231**	Sly	Pore-form toxin	Invasion	Unaffected-pig	[38]
**Histidine peptidase-SS1215**	PepD	Subtilisin- protease	Invasion	No mutant	[21]
**Anchored DNA nuclease-SSU1760**	SsnA	Host DNA degradation	Invasion	Not tested	[39]
**Cell envelope proteinase-SSU0757**	SspA	Subtilisin- protease	Invasion	Attenuated-mouse	[40]

No mutant = No gene knock-out mutant strain published; Not tested = Virulence factor revealed by the construction of gene knock-out mutant, whose mutant strain has not been tested for virulence in animal models

^1^Gbs = globoseries glycolipids including globotriaosylceramide (e.g.: Gb3/CD77)

^2^ECM = Extra Cellular Matrix;

^3^GAG = host cell surface glycosaminoglycan

### Variation of SadP protein sequences correlate with *S*. *suis* genotype

**Fig 2:**
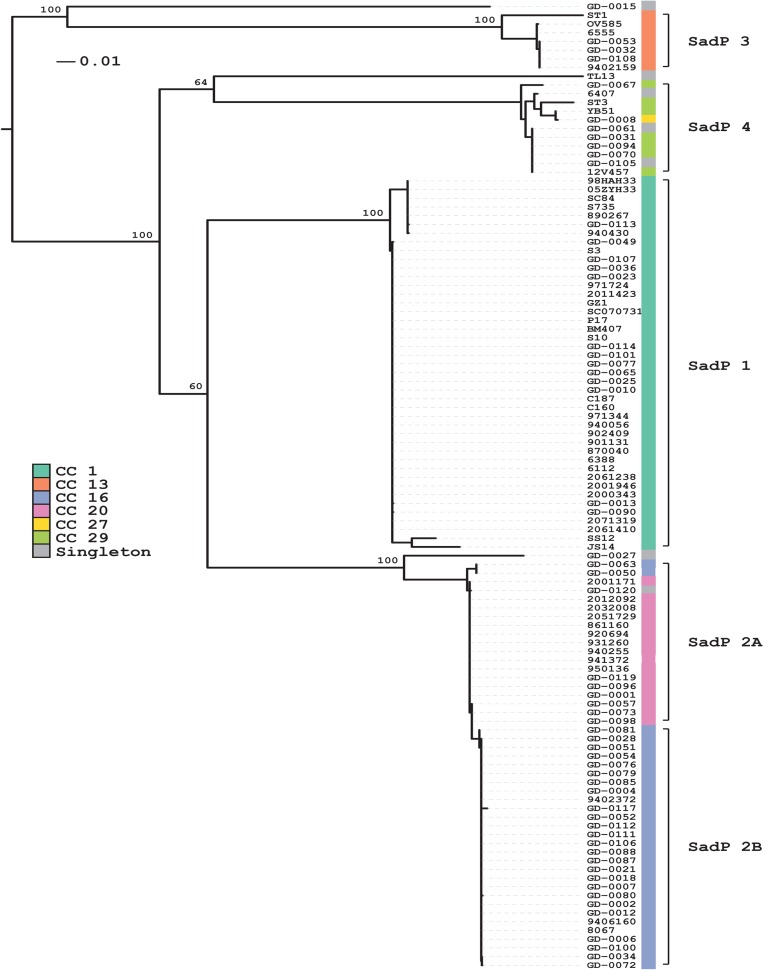
Sequence analysis of SadP of 111 invasive strains isolated from humans and pigs, revealed substantial variation across different serotypes and genotypes. *SadP* protein sequences clustered according to *S*. *suis* MLST clonal complexes (CC) genotype. Variants of SadP were identified in 111 out of 116 strains covering CC1, CC13, CC16, CC20 and CC27/29. SadP1 was present in zoonotic CC1 strains and was consistently present across serotype 2 isolates, whilst SadP2A and Sad2B were found in the zoonotic CC20 and non-zoonotic CC16 strains, respectively. Colored blocks indicate to which clonal complex a strain belongs and the range of each of the SadP variants is indicated with brackets.

**Fig 3:**
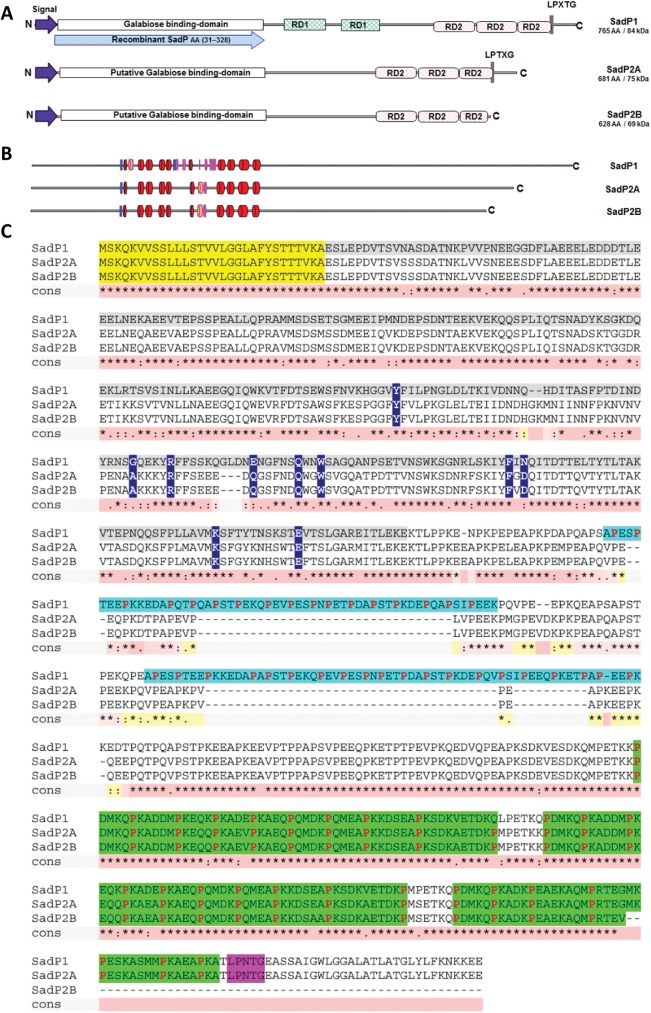
Structural comparison of SadP proteins and their subdomains in different *S*. *suis* strains. (A) Schematic diagram of the domain organization of SadP in the primary sequence. The N-terminal domain (residues 55–200) includes the galabiose-binding domain (the blue arrow indicates the recombinant protein (31–328) of the N-terminal domain derived from SadP1 that showed high-affinity binding to the terminal-epitope galabiose of the Gb3/CD77 host-cell receptor. The C-terminal domain (residues 418–472; red), which should have H-factor-binding property, contains a Repeat Domain (RD) rich in proline [34]. The blue box indicates the YSIRK-type signal peptide (residues 5–30), and the black box indicates the LPXTG motif (residues 524–528) present only in SadP1 and SadP2A. (B) Schematic secondary structure solved by crystal structure analysis of the N-terminal galabiose-binding domain of SadP1 (PDB: 5BOB from 139–323 AA) (α-helix in red, 3/10 α-helix in fuchsia, β-sheets in blue). The secondary structures of SadP2A and SadP2B were predicted according to the solved structure of SadP1 deposited in http://www.rcsb.org/pdb/explore/remediatedSequence.do?structureId=5BOB. (C) Comparison of primary AA-sequences of SadP1, SadP2A and SadP2B variants and the consensus sequence density. Sequences highlighted correspond to the secreted signal sequence (yellow), and the first and second series of repeat domains RD1 (cyan) and RD2 (green). RD1 is only present in SadP1 whilst the gram-positive anchor LPXTG motif is present only in SadP1 and SadP2A (magenta). The degree of consensus sequence density is indicated in pink. The galabiose-binding domain (31–328 AA in grey) showed the most variable AA-sequence among the three different variants (SadP1 vs SadP2A 69.9%, SadP1 vs SadP2B 69.1%, SadP2A vs SadP2B 99.3%). In the galabiose-binding domain, the binding sites versus the Gal1-4βGal of Gb2, an analog of Gb3/CD77, are indicated in dark blue [37]. The C-terminal region included two series of **P**ro-rich (**P**roline) Repeats Domains (RD) of a maximum of 66 AA (RD1) and 54 AA sequence (RD2) length. The number and length of repeats located in the C-terminal domain also varied within SadPs variants; in particular, SadP1 possessed a higher number of RD (first series: RD1-RD1; second series: RD2-RD2-RD2) which explains the observed size variation across SadP variants.

To determine the contribution of SadP to the bacteria-IEC interaction, we investigated whether genetic variation of SadP occurred among zoonotic and non-zoonotic isolates with different serotypes and genotypes. We detected five genetic variants of SadP in 111 out of 116 strains covering CC1, CC13, CC16, CC20, and CC27/29. The clustering of the five genetic variants occurred independent of the serotype but according to their clonal complex (**Fig 2**). We designated the variants SadP1 (CC1), SadP2A (CC20), SadP2B (CC16), SadP3 (CC13) and SadP4 (CC27/29). An expanded search including an additional 375 strains from Vietnam and the UK [29] demonstrated that these five variants are likely to cover the vast majority (94%) of SadP variants present in *S*. *suis* (**Fig A in S1 File**). At the amino acid (AA) level, SadP1 shares a similarity of 79% and 77% with SadP2A and SadP2B respectively. SadP2A and SadP2B exhibit a 99% similarity in AA-sequence and differ by the absence of the LPXTG cell-anchor motif in SadP2B, which suggests SadP2B might be secreted instead of anchored to the cell wall (**Fig 3A** and **Fig 3C**). The partial crystal structure of the galabiose-binding domain of the SadP1 adhesin (from 139–323 AA) has recently been resolved (PDB: 5BOA) [41]. The predicted secondary structures of SadP2A and SadP2B possess fewer α-helices, compared to SadP1 (**Fig 3B**).

### Different forms of SadP vary in their contributions to bacterial adhesion to IEC

**Fig 4:**
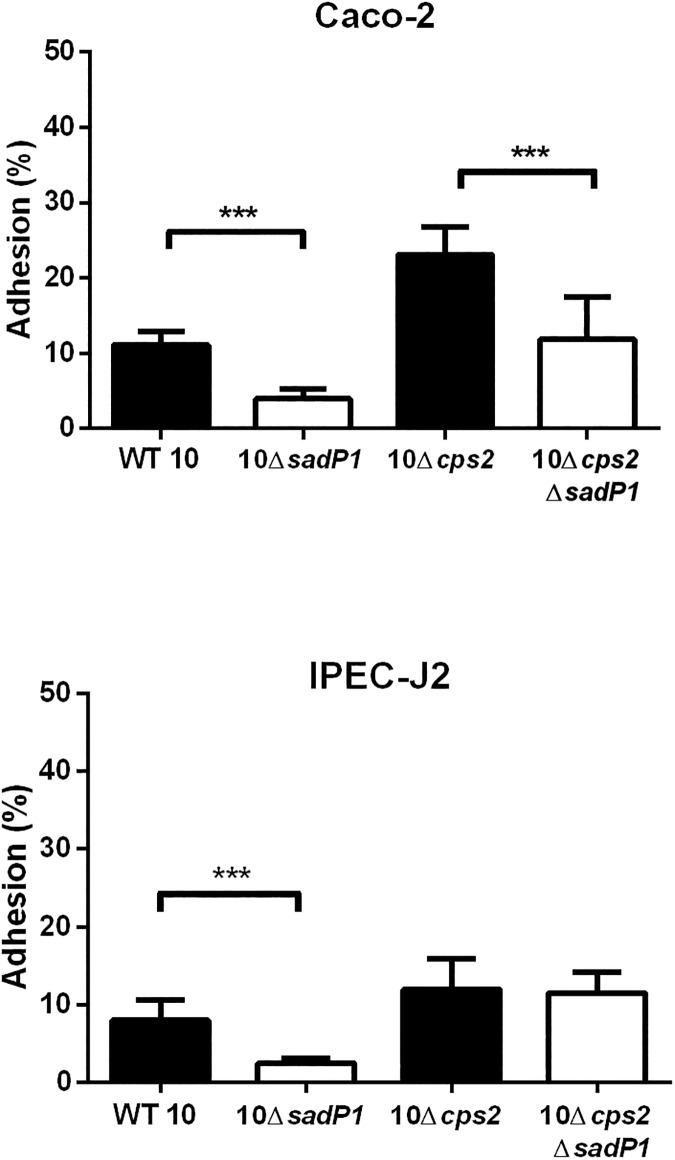
SadP1 contributes to the adhesion of human and porcine IEC. Adhesion to human (Caco-2) and porcine (IPEC-J2) IEC of WT 10 strain of SS2/CC1 and unencapsulated 10Δ*cps*2 mutant and their isogenic *sadP* mutants (10Δ*sadP*1 and 10Δ*cps2*Δ*sadP1* respectively). Adhesion was expressed as the total number of IEC associated bacteria, including both adherent and intracellular bacteria, proportional to the total number of bacteria added to the IEC infection model. Three independent experiments (i.e.: biological replicates) were performed in triplicate and the combined together. Unpaired Student's t-test was used to compare each Δ*sadP*1 mutants with its own parental strains (***, *p*< 0.001; **, *p*< 0.01; *, *p*< 0.05).

Next, we evaluated the contribution of SadP variants to adhesion to human and porcine IEC. We focused on SadP1 and two subvariants of SadP2 (SadP2A and SadP2B) as these variants were present in the most common zoonotic SS2/CC1 (SadP1) and SS2/CC20 (SadP2A) and non-zoonotic SS9/CC16 (SadP2B) strains (**Fig 2**). We deleted *sadP1* in SS2/CC1 strain 10 and its unencapsulated mutant 10Δ*cps*2 (10Δ*sadP1* and 10Δ*cps2*Δ*sadP1* respectively). Adhesion was expressed as the total number of IEC associated bacteria, including both adherent and intracellular bacteria, proportional to the total number of bacteria added to the IEC infection model, since we have previously shown that under the test conditions the number of invasive bacteria is very low (from 0.04% to 0.01% of the starting inocolum) and can be neglected [6]. 10Δ*sadP1* showed a two-fold reduction in adhesion to Caco-2 as well as to IPEC-J2 cells compared to the parental strain indicating that SadP1 contributes to the adhesion to both human and porcine IEC (*p*<0.001, **Fig 4**). The double knockout 10Δ*cps2*Δ*sadP1* also showed a two-fold reduction in adhesion compared to parental 10Δ*cps*2 strain (*p*<0.001, **Fig 4**). However, no significant reduction in adhesion was observed when we tested 10Δ*cps2*Δ*sadP1* in contact with IPEC-J2 cells. Finally, we created *sadP* knockouts in SS9/CC16 strain 8067 (8067Δ*sadP2B*) and SS2/CC20 strain 2001171 (2001171Δ*sadP2A)* to test the adhesion properties of SadP2A and SadP2B in their natural genetic background. Adhesion capacity of both 2001171Δ*sadP2A* and 8067Δ*sadP2B* was similar compared to their parental strains when brought into contact with Caco-2 cells. In contrast, 8067Δs*adP2B* adhered significantly less (1.6-fold) to the IPEC-J2 cells than the parental strain 8067 WT (*p*<0.01) indicating that SadP2B in SS9/CC16 strain 8067 contributed to the adhesion to porcine IEC (**Fig B in S1 File**).

### Complementation of *sadP1* gene function with different of genetic variants of *sadP*

**Fig 5:**
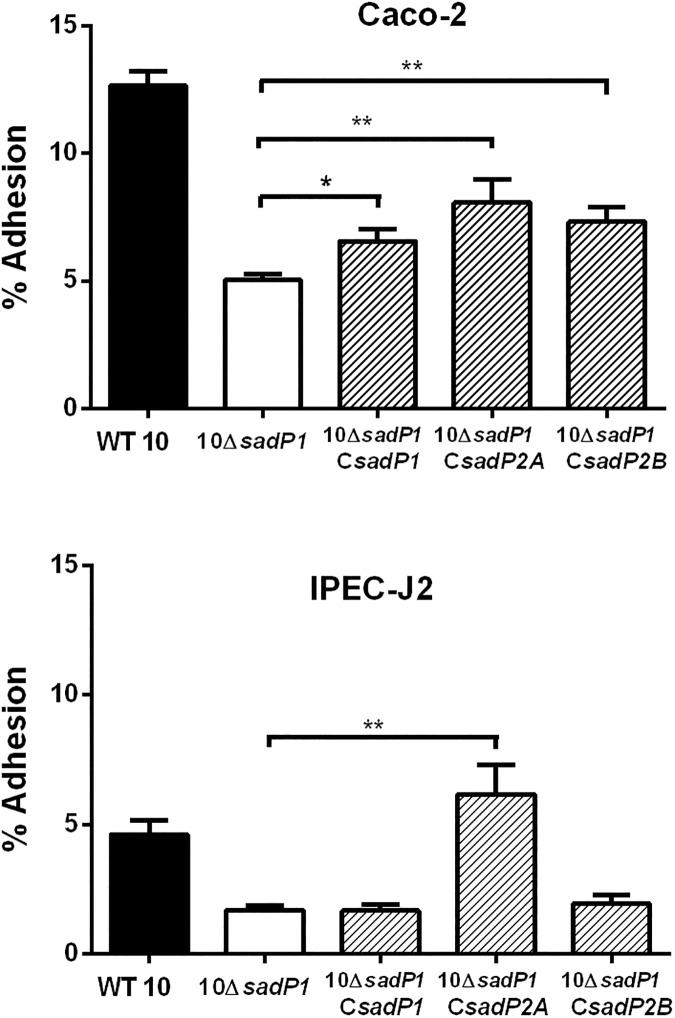
Adhesion of *S*. *suis* Δ*sadP1* deletion mutants and Δ*sadP1* mutants complemented with different *sadP* variants, to human and porcine IEC. Influence of SadP variants on adhesion of strain 10 of SS2/CC1 genetic background to human (Caco-2) and porcine (IPEC-J2) cells. All strains carry either an empty pMX1 (WT) or with one of three SadP variants cloned into pMX1 carrying a *spc*^*r*^ gene. Results were determined after 2h of co-incubation with IECs at 37°C. Three independent experiments were performed in triplicate or quadruplicate and the combined together. Unpaired Student's t-test was used to compare each 10Δ*sadP1* mutant or complemented mutants with its own parental strain (***, *p*< 0.001; **, *p*< 0.01; *, *p*< 0.05).

Complementation mutants were subsequently created by sub-cloning variants of with three different sadP (*sadP1*, *sadP*2*A* and *sadP2B*) in the 10Δ*sadP1* with SS2/CC1 background. Growth kinetics did not differ significantly between the wild-type (WT) strain, 10Δ*sadP1* and complementation mutants, and transcripts of each *sadP* variant were detected by qPCR (data not shown). Adhesion to Caco-2 cells was increased by expression of any of the three *sadP* variants in 10Δ*sadP1*, although not to WT levels (**Fig 5**). Adhesion of 10Δ*sadP1*C*sadP1* to Caco-2 cells was 1.2-fold higher than that of 10Δ*sadP*1 (*p* = 0.057), but adhesion to IPEC-J2 cells was similar to that of the knockout. However, adhesion to Caco-2 cells of 10Δ*sadP1*C*sadP2A* was 1.4-fold higher (*p* = 0.02) and 10Δ*sadP*1Cs*adP*2B was 1.3-fold higher (*p* = 0.004) than that of 10Δ*sadP1*. Only 10Δ*sadP1*C*sadP*2A was able to restore adhesion to IPEC-J2 cells, which was 3-fold higher than that of the 10Δ*sadP1* (*p* = 0.003) (**Fig 5**).

### *S*. *suis* adhesion to human IEC is inhibited by anti-Gb3/CD77

**Fig 6:**
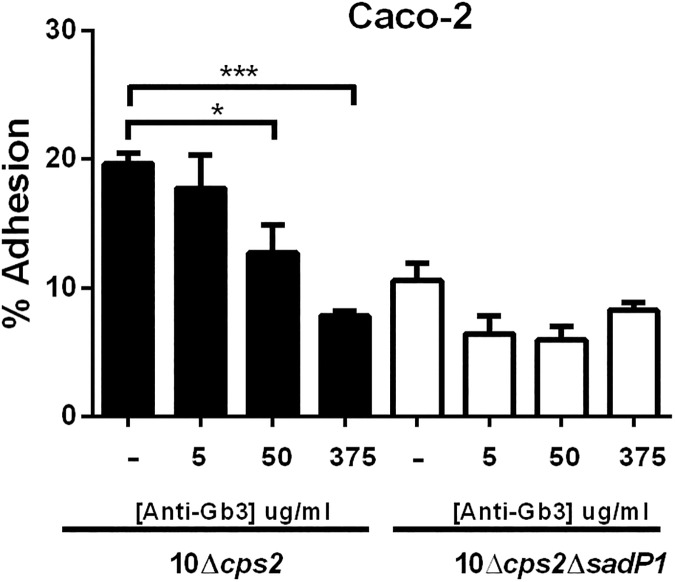
SadP1 contributes to adhesion to human IEC by binding the Gb3/CD77 receptor. Dose-dependent competitive inhibition of human IEC (Caco-2) cells by anti-Gb3/CD77 antibody for SS2/CC1 10Δ*cps2* and SS2/CC1 10Δ*cps2*Δ*sadP1*. Unpaired Student's t-tests were used to test significant different adhesion percentages compared to the parental strains (***, *p*< 0.001; **, *p*< 0.01; *, *p*< 0.05).

To further investigate if adhesion is indeed mediated through binding of SadP1 to Gb3/CD77, we performed an inhibition assay with anti-Gb3/CD77. The inhibition of adhesion by strain 10Δ*cps*2 was compared with the unencapsulated mutant lacking SadP1 function (10Δ*cps2*Δ*sadP1*) since this mutant showed dramatically reduced adhesion (approx. 2.0-fold, *p*<0.001) compared to its parental unencapsulated strain (10Δ*cps2*). The adhesion of 10Δ*cps2* to human IEC was inhibited by anti-Gb3/CD77 in a dose-dependent manner while no significant inhibition effect could be observed with the double mutant 10Δ*cps2*Δ*sadP1* (**Fig 6**).

### SadP1 is involved in *S*. *suis* translocation across human intestinal epithelial cells

**Fig 7:**
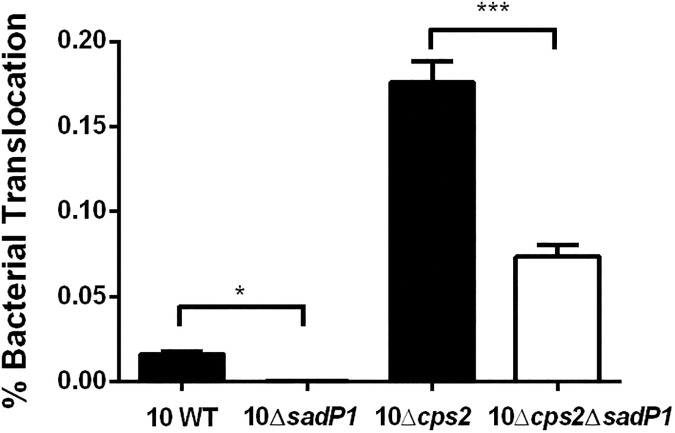
SadP1 contributes to the translocation of *S*. *suis* SS2/CC1 across human IEC. Comparison of bacterial translocation efficiency of Δ*sadP1* mutant strains and their encapsulated and unencapsulated (WT 10 and 10Δ*cps2*) parental strains across differentiated human IEC (Caco-2). Unpaired Student's t-tests were used to test significant different translocation percentages compared to the parental strains (***, *p*< 0.001; **, *p*< 0.01; *, *p*< 0.05).

To test whether SadP1 might have an effect on the translocation across the intestinal epithelium, we tested the 10Δ*sadP1* and 10Δ*cps2*Δ*sadP1* mutants and their parental strains for their translocation capacity across polarized Caco-2 cells [6]. We observed a significant difference in translocation between strains with and without SadP1, in particular between unencapsulated mutant 10Δ*cps2* and its isogenic 10Δ*cps2*Δ*sadP1* mutant (2.3-fold, *p*<0.001) (**Fig 7**).

## Discussion

Similar to other streptococcal surface proteins involved in a wide range of physiological functions [42], SadP represents a bifunctional protein with host-cell adhesion and host immune evasion properties [43–46]. The C-terminal domain of SadP was reported to bind to human complement factor H (Fhb), which is a glycoprotein that regulates the complement activation [43]. The galabiose-binding N-terminal domain of SadP showed high-affinity and specific binding to the Gb3/CD77 host-cell receptor [11, 47].

Transcript levels of SadP were highly up-regulated compared to transcript levels of other virulence factors in zoonotic and non-zoonotic isolates when interacting with human and porcine IEC, although SadP transcript levels varied between strains. Thus, we hypothesized that SadP is involved in the interaction with host IEC. We identified five main SadP genetic variants among 116 zoonotic and non-zoonotic isolates, which clustered according to genotype. We focused on a comparison of three SadP variants present in the most virulent genotypes circulating in the Netherlands: CC1, CC16 and CC20. In particular, SadP1 was associated to zoonotic and virulent isolates with CC1 genetic background.

We observed differences in the ability of SadP to adhere to IEC between *S*. *suis* strains carrying different SadP variants. The SadP1 deletion mutant of SS2/CC1 encapsulated strain 10 showed significantly lower adhesion to human as well as to porcine IEC than the parent strain. Previously it has been demonstrated that the expression of the *cps* could change in response to environmental stimuli *in vivo* [21, 48] and the presence or thickness of the capsule affected the bacterial interaction with the host-cells [6, 10]. Therefore, we studied the effect of SadP1 on IEC adhesion in SS2/CC1 10 strain unencapsulated mutant (10Δ*cps2*), in which bacterial adhesins could have better access to bind specific host-cell receptors. The double 10Δ*cps2*Δ*sadP1* mutant still showed a significant reduction in adhesion to human IEC compared to its parental mutant but did not show a significant reduction in binding to porcine IEC. These results suggest that SadP1 strongly promotes the bacterial adhesion to the human intestinal epithelium independent of CPS expression, and contributes to the zoonotic potential of SS2/CC1 strains that mostly carry the SadP1 variant. Although the complemented SadP1 failed the full restoration of adhesion activity of the wild-type phenotype, its adhesion capacity with human IEC was significantly increased compared to the deletion mutant Δ*sadP1*. The somewhat lower adhesion of the complemented SadP1 may have been due to a low *sadP1* gene expression or due to a suboptimal folding and assembly of SadP1 protein on the bacterial cell wall in our complementation system thus interfering with a correct docking of the SadP adhesin onto the galabiose moiety of the Gb3/CD77 receptor.

Interestingly, both encapsulated and unencapsulated SadP1 mutants were also impaired in their translocation across human IEC, supporting the hypothesis that direct adhesion and interaction of bacteria with specific host-cell receptors, potentially leading to the opening of the cellular tight junctions, is required for bacterial translocation [16]. Thus, we postulate that SadP1 contributes to colonization of human intestinal epithelium and hence permits *S*. *suis* translocation across the intestinal mucosa after adhesion. However, SadP1 deletion does not entirely abolish the bacterial adhesion capacity, similar to observations from other investigators when studying *S*. *suis* Fhb-mediated adhesion to epithelial and endothelial cells originating from other organ systems [45], indicating that other bacterial surface-associated factors are also likely to contribute to the adhesion of *S*. *suis* [16].

SadP1 and SadP2 notably differ in AA composition (77% similarity), while SadP2A and SadP2B share 99% similarity and differ only for the lack of LPXTG cell-anchor motif in SadP2B. The lack of this motif might suggest that SadP2B will be secreted instead of being anchored to the cell wall. We have previously shown that the zoonotic SS2/CC20 clone emerged from the non-zoonotic SS9/CC16 clone and the sequence similarity (99%) of the SadP2 variants present in CC20 and CC16 isolates is consistent with this observation [23]. While deletion of *sadP2B* in an SS9/CC16 strain resulted in a reduction of adhesion to porcine IEC, deletion of *sadP2A* in an SS2/CC20 strain had no effect on adhesion to both human and pig IEC. However, complementation with SadP2A, but not with SadP2B, restored the adhesion capacity to human (*p*< 0.05) and particularly to porcine IEC (*p*< 0.01) of Δ*sadP1* in a SS2/CC1 strain. Thus, in the SS2/CC20 strain, other factors than SadP may contribute to adhesion masking the effect of SadP2A deletion, while the LPXTG cell-anchor motif present in SadP2A, but absent in SadP2B, might be a prerequisite for SadP mediated adhesion of CC1 to porcine IEC.

These results suggest that SadP2 variant is involved in the adhesion of IEC, but with a different degree of “host-specificity’ than SadP1, since it seems to mediate a major effect in the binding with porcine IEC.

We demonstrated that SadP1 contributes to the adhesion to human IEC by binding of Gb3/CD77 confirming previous functional studies on recombinant galabiose-binding N-terminal domain of SadP recognizing Gb3/CD77 as its natural glycolipid host-cell receptor [11, 47]. Recent studies on the structure of SadP1 in interaction with Gb2, which is analogous to Gb3/CD77, identified 10 AA critical for binding of SadP1 to the homologous structure of galabiose of Gb2 [47]. AA alignment of SadP1 and the two SadP2 variants revealed three relevant AA substitutions (Gly_233_→Ala_233_, Glu_249_→Gln_249,_ Asn_285_→Asp_285_) in the binding pocket of the galabiose. These three AA are critical for the binding specificity with α-D-Gal of Gb2, and these substitutions might result in differences in binding affinity to different isoforms and forms of Gbs receptors among the SadP variants. This observation would be analogous to Stx variants, associated with different clinical outcomes of infections, which bind various isoforms and forms of Gbs receptors with different affinity as determined by variation in fatty acid composition [49–52]. Similarly, differences in receptor recognition of Stx variants are known to mediate host-specificity [53, 54]. Gbs receptors, including Gb3/CD77 and Gb4, are widely expressed in tissues including human and porcine intestine [13, 14, 51]. Nevertheless, the distribution of these receptors varies between species, individuals and populations [12, 51]. With rare exceptions, erythrocytes of all individuals express different isoforms of Gbs receptors. Interestingly, erythrocytes with high Gb3/CD77 levels predominate (69–75%) in areas of Southeast Asia compared to other populations [12]. It is tempting to speculate that Southeast Asian population may be more susceptible to SS2/CC1 infection due to the high prevalence of the Gb3/CD77 receptor, which could, in addition to differences in exposure, partly explain the much higher *S*. *suis* disease incidence in South-East Asia [2].

Our findings contribute to a better understanding of specific host-pathogen interactions, showing that *S*. *suis* SadP1 contributes to the adhesion and translocation across host IEC. Different SadP variants might contribute to differences in host restriction between *S*. *suis* clones, due to differences in affinity and specificity of SadP-Gbs receptors interactions. Thus, components that interfere with bacterial binding to these receptors may have potential to reduce the burden of *S*. *suis* infection in both pigs and humans.

## Supporting information

S1 File Fig A: Phylogenetic analysis of SadP variants including strains from Vietnam and the UK. The five identified SadP variants cluster together even when 375 strains from Weinert *et al*. [29] were included in the analysis, suggesting that the proposed five variants accurately describe the globally present SadP protein variants. The 111 strains used for determination of the variants are highlighted with colored blocks indicating to which clonal complex the strain belongs. Brackets indicate the different SadP variants. Fig B: Adhesion of *S*. *suis* Δ*sadP2A and* Δ*sadP2B* deletion mutants to human and porcine IEC. Percentage of adhesion of strain SS2/CC20 2001171 WT and its 2001171Δ*sadP*2A mutant; SS9/CC16 8067 WT strain and its isogenic mutant Δ*sadP*2B (8067Δ*sadP*2B) to human (Caco-2) and porcine (IPEC-J2) intestinal cells. Three independent experiments were performed in triplicate and the combined together. Unpaired Student's t-test was used to compare each Δ*sadP*2A and Δ*sadP*2B mutants with its own parental strains (***, *p*< 0.001; **, *p*< 0.01; *, *p*< 0.05). Table A: List of *S*. *suis* strains used in this study. Table B: List of primers used in this study.
(DOCX)
