# Supplementary material for: Streptococcal Adhesin P (SadP) contributes to Streptococcus suis adhesion to the human intestinal epithelium
Source: PLoS One. 2017 Apr 13;12(4):e0175639. doi: 10.1371/journal.pone.0175639 (PMC5391093; doi:10.1371/journal.pone.0175639)
Supplement: S1 File — Fig A: Phylogenetic analysis of SadP variants including strains from Vietnam and the UK. The five identified SadP variants cluster together even when 375 strains from Weinert et al. [29] were included in the analysis, suggesting that the proposed five variants accurately describe the globally present SadP protein variants. The 111 strains used for determination of the variants are highlighted with colored blocks indicating to which clonal complex the strain belongs. Brackets indicate the different SadP variants. Fig B: Adhesion of S. suis ΔsadP2A and ΔsadP2B deletion mutants to human and porcine IEC. Percentage of adhesion of strain SS2/CC20 2001171 WT and its 2001171ΔsadP2A mutant; SS9/CC16 8067 WT strain and its isogenic mutant ΔsadP2B (8067ΔsadP2B) to human (Caco-2) and porcine (IPEC-J2) intestinal cells. Three independent experiments were performed in triplicate and the combined together. Unpaired Student's t-test was used to compare each ΔsadP2A and ΔsadP2B mutants with its own parental strains (***, p< 0.001; **, p< 0.01; *, p< 0.05). Table A: List of S. suis strains used in this study. Table B: List of primers used in this study. (DOCX) [file pone.0175639.s001.docx]

**S1 File:**

**Fig A. Phylogenetic analysis of SadP variants including strains from Vietnam and the UK.**

The five identified SadP variants cluster together even when 375 strains from Weinert *et al*. [[29](#_ENREF_29)] were included in the analysis, suggesting that the proposed five variants accurately describe the globally present SadP protein variants. The 111 strains used for determination of the variants are highlighted with colored blocks indicating to which clonal complex the strain belongs. Brackets indicate the different SadP variants.

**
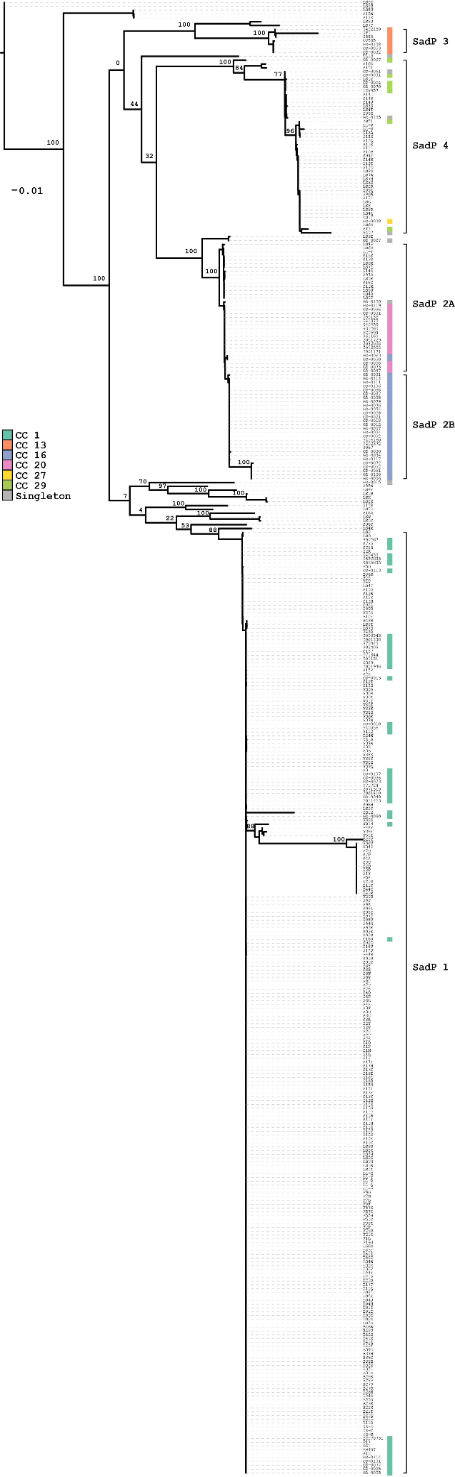
**

**Fig B. Adhesion of *S. suis*** Δ***sadP2A and*** Δ***sadP2B* deletion mutants to human and porcine IEC.**

Percentage of adhesion of strain SS2/CC20 2001171 WT and its 2001171Δ*sadP*2A mutant; SS9/CC16 8067 WT strain and its isogenic mutant Δ*sadP*2B (8067Δ*sadP*2B) to human (Caco-2) and porcine (IPEC-J2) intestinal cells. Three independent experiments were performed in triplicate and the combined together. Unpaired Student's t-test was used to compare each Δ*sadP*2A and Δ*sadP*2B mutants with its own parental strains (***, *p*< 0.001; **, p< 0.01; *, p< 0.05).

**

**

**Table A. List of *S. suis* strains used in this study.**

**Table A**

| ***S. suis* isolates** | **Serotype** | **Sequence Type** | **Clonal Complex** | | **Clinical source** | | **% Adhesion^1^ Human IEC** | | | **% Adhesion^1^ Porcine IEC** | | **Ref.** | |
| --- | --- | --- | --- | --- | --- | --- | --- | --- | --- | --- | --- | --- | --- |
|  |  |  |  |  |  |  |  |  |  |  |  |  |  |
| 10 | 2 | 1 | 1 | | Pig meningitis | | | 12.6 ± 1.2 | | 6.6 ± 0.3 | | [[5](#_ENREF_5), [41](#_ENREF_41)] | |
| 8067 | 9 | 136 | 16 | | Pig meningitis | | | 8.3 ± 1.1 | | 15.5 ± 5.8 | | [[5](#_ENREF_5)] | |
| 2001171 | 2 | 20 | 20 | | Human meningitis | | | 29.4 ± 3.5 | | 14.0 ± 2.4 | | [[5](#_ENREF_5)] | |
| **Unencapsulated mutant** | |  |  |  | |  | | |  | |  | |  |
| 10Δ*cps*2 | 2 | 1 | 1 | |  | | | 31.1 ± 3.5 | | 12.5 ± 1.0 | | [[42](#_ENREF_42)] | |

| **Mutants** | **WT** | | **Knock-out gene** | | **Description** | |  | |
| --- | --- | --- | --- | --- | --- | --- | --- | --- |
| 10Δ*sadP1* | 10 | | | *sadP1* | *Janus* inserted into *sadP*1 | | This work | |
| 10Δ*cps*2 | 10 | | | *cpsEF* | *spc* inserted into *cpsEF* | | [[25]](file:///C:\Users\Laura\Dropbox\Mutants%20SadP\Article\Table%201.xlsx#RANGE!_ENREF_25) | |
| 10Δ*cps*2Δ*sadP1* | 10 | | | *cpsEF*-*sadP1* | *spc* inserted into *cpsEF*, *Janus* inserted into *sadP1* | | This work | |
| 10Δ*sadP*1C*sadP1* | 10 | | | *sadP1* | Complementation of *sadP1* | | This work | |
| 10Δ*sadP*1C*sadP2A* | 10 | | | *sadP1* | Complementation of *sadP2A* | | This work | |
| 10Δ*sadP*1C*sadP2B* | 10 | | | *sadP1* | Complementation of *sadP2B* | | This work | |
| 2001171Δ*sadP2A* | 2001171 | | | *sadP2A* | *Janus* inserted into *sadP2A* | | This work | |
| 8067Δ*sadP2B* | 8067 | | | *sadP2B* | *Janus* inserted into *sadP2B* | | This work | |
| **Plasmid** | |  | |  | **Description** |  |  | |
| pMX1 | |  | |  | *spc*^r^, pSSU1 *ori*, *S. suis malX* promoter, the derivative of pSET2 | | | [[28](#_ENREF_28)] |

^1^ Adhesion average ± Standard Deviation of at least two independent experiments performed in triple.

**Table B. List of primers used in this study.**

**Table B**

| **Real time qPCR^1^** | | **Gene from SS2/CC1 strain P1/7 (SSU locus) or SS9/CC16 strain 8067** | | | | **Primer** | **Sequence (5'-->3')** |
| --- | --- | --- | --- | --- | --- | --- | --- |
| *proS* (reference) | | Proline tRNA transferase (SSU1753) | | | | *proS*_F | AATGGCAACTAGCGAATACA |
|  |  |  |  |  |  | *proS*_R | ACCAACTACAGCGTTCTTCA |
| *gdh2* (reference) | | Glutamate dehydrogenase of SS2/CC1 (SSU0234) | | | | *gdh2*_F | AGCCACACAGTACACGC |
|  |  |  |  |  |  | *gdh2*_R | TTACGGTTTGGTTTACTTCACT |
| *gdh9* (reference) | | Glutamate dehydrogenase of SS9/CC16 | | | | *gdh9*_F | GTTGGCAGCAAACGGTA |
|  |  |  |  |  |  | *gdh9*_R | ACCCTCTGCCACACAGTAAAC |
| *sly* (target) | | Suilysin (SSU1231) | | | | *sly*_F | ATTGATAATCCGCCAGC |
|  |  |  |  |  |  | *sly*_R | TACTGTATGCCATTGTTTCATC |
| *sadP*1 (target) | | Streptococcal adhesin P1 of SS2/CC1 (SSU0253) | | | | *sadP1*_F | TTGATACAAGCGAATGGTCTTT |
|  |  |  |  |  |  | *sadP1*_R | CTGCCAGAAGAGGGAATGA |
| *sadP2B* (target) | | Streptococcal adhesin P2B of SS9/CC16 | | | | *sadP2B*_F | CGGAAGAAGGTCAGATTCA |
|  |  |  |  |  |  | *sadP2B*_R | TGACGCAGTCACTTTAGCA |
| *neuB* (target) | | N-acetylneuramic acid synthase (SSU0535) | | | | *neuB*_F | ACTGGTATGGCTGTTATGGA |
|  |  |  |  |  |  | *neuB*_R | TTCTTCTGGCTCTTTTTCAA |
| *cpsF*2 (target) | | Galactosyl rhamnosyl transferase­­*_cpsF*2 (SSU0520) | | | | *cpsF*_F | TGCTTGTCGGATTGGTCCTT |
|  |  |  |  |  |  | *cpsF*_R | CAGGCACAAATCGTCCAACA |
| *cpsE*9 (target) | | Galactosyl_transferase_*cps9E* | | | | *cpsE*9_F | GGCTACCAATACACGCTTTAT |
|  |  |  |  |  |  | *cpsE*9_R | CGCTTCTAGGAGACTAGGAT |
| *eno* (target) | | Enolase (SSU1320) | | | | *eno*_F | TGCCAAGACGCTCAGTA |
|  |  |  |  |  |  | *eno*_R | AGTATTCCACGCTTTGAAGA |
| *dppIV* (target) | | Di-peptidyl peptidase IV (SSU0187) | | | | *dppIV* _F | TTTGCCATTGAGAATTATCC |
|  |  |  |  |  |  | *dppIV* _R | AACTCCGTGTCGCTTCTA |
| *HP0197/hepIII* (target) | | Heparinase II/III-like protein (SSU1048) | | | | *HP0197* _F | GTTCAGTCGGGTATCATCTT |
|  |  |  |  |  |  | *HP0197*_R | TCTTTGGCACGGTAATG |
| *ssnA* (target) | | Surface-anchored DNA nuclease (SSU1760) | | | | ssnA _F | CCCTGTTGTCACCTCTTC |
|  |  |  |  |  |  | ssnA _R | TGTGGCATCGGTATCAATA |
| **Generation of mutants** | **Function** | | | **Primer** | **Sequence (5'-->3') ^2^** | | |
| 10Δ*sadP1* | Left PCR fragment | | *sadP*2_F | | GCAAGCAGAAAGTTGTGTCCAG | | |
|  | Left PCR fragment | | *sadP*2R_*ApaI* | | ACTGA***GGGCCC***TGACCTTCTTCCGCCTTCA | | |
| 10Δ*cps2*Δ*sadP1* | Right PCR fragment | | *sadP*2F_*BamHI* | | TAAGT***GGATCC***AAAACAAGAAGATGTTCAACCG | | |
|  | Right PCR fragment | | *sadP*2_R | | CTTCATACCCTCTGTCCGTG | | |
| 2001171Δ*sadP2A* | Left PCR fragment | | *sadP*9_F | | CAGTAGGTGAGTGGGAAAATGT | | |
|  | Left PCR fragment | | *sadP*9R_*ApaI* | | ACTGAGGGCCCTCTCTATCACCGCCAGTCTTA | | |
| 8067Δ*sadP2B* | Right PCR fragment | | *sadP*9F_*BamHI* | | TAAGT***GGATCC***CCGAAGTTCCAAAGGCAG | | |
|  | Right PCR fragment | | *sadP*9_R | | CTGTGGCAAGAGTGGCTAAAG | | |
| 10Δ*sadP*1C*sadP1* | PRC SadP1 from SS2/CC1 | | *cSadP1F_BamHI* | | GTTCTT***GGATCC***ATTTAAGAAGGAGCCAATTATGAG | | |
|  |  |  | *cSadP1R_EcoRI* | | TCAGT***GAATTC***CTATTCTTCTTTTTTGTTTTTGAATAG | | |
| 10Δ*sadP*1C*sadP2A* | PCR SadP2A from SS2/CC20 | | *cSadP2A_F_BamHI* | | GTTCTT***GGATCC***ATTTAAGAAGGAGCCAATTATGA | | |
|  |  |  | *cSadP2A_R_EcoRI* | | TCAGT***GAATTC***CTATTCTTCTTTTTTATTTTTGAATAGAT | | |
| 10Δ*sadP*1C*sadP2B* | PCR SadP2B from SS9/CC16 | | *cSadP2B_F_BamHI* | | GTTCTT***GGATCC***ATTTAAGAAGGAGCCAATTATGAGC | | |
|  |  |  | *cSadP2B_R_EcoRI* | | TCAGT***GAATTC***TCATACCTCTGTTCGTGGCAT | | |
| **Confirmation of mutants** | | | |  |  | | |
|  | Janus cassette | | | Janus_F | CTTTAAATACTGTAGAAAAG | | |
|  | Janus cassette | | | Janus_R | ACCACGCTGTGCTCTTGC | | |

**^1^** Primers for qRT-PCR analyses of gene transcript expression, including the reference genes *proS* and *gdh*

**^2^** ApaI (***GGGCCC***)/BamHI (***GGATCC***)/EcoRI (***GAATTC***)-tagged primers for generation of mutant
